# Editorial: Abiotic stress and plant immunity – a challenge in climate change

**DOI:** 10.3389/fpls.2023.1197435

**Published:** 2023-05-10

**Authors:** Kiwamu Tanaka, Yashwanti Mudgil, Meral Tunc-Ozdemir

**Affiliations:** ^1^ Department of Plant Pathology, Washington State University, Pullman, WA, United States; ^2^ Department of Botany, University of Delhi, New Delhi, India; ^3^ American College of Healthcare Sciences, Portland, OR, United States

**Keywords:** plant immunity, global warming, climate change, biotic stress and abiotic stress, disease triangle, biotic stress, abiotic stress

Plants are constantly under various environmental pressure in nature, which affects their growth, reproduction, yield, and survival. Global warming and climate change have aggravated background stress levels, making plant response to stress combinations a pressing concern (Mora et al., 2015; Mankin et al., 2019). In the coming decades, the geographical areas suitable for growing certain plants are likely to undergo significant changes as a result of varying greenhouse gas and aerosol emission scenarios (**Figure 1** provides a specific example in the US). Plants need to sense, sort, and communicate multiple stress signals and then activate downstream responses while simultaneously allocating resources. Thus, the response to multiple stress exposure needs to be studied to tackle the grand challenge of climate change. In this Research Topic issue, several important aspects of abiotic stress and plant immunity have been covered, which could provide some hints to cope with the extreme challenges of feeding the growing world population.

**Figure 1:**
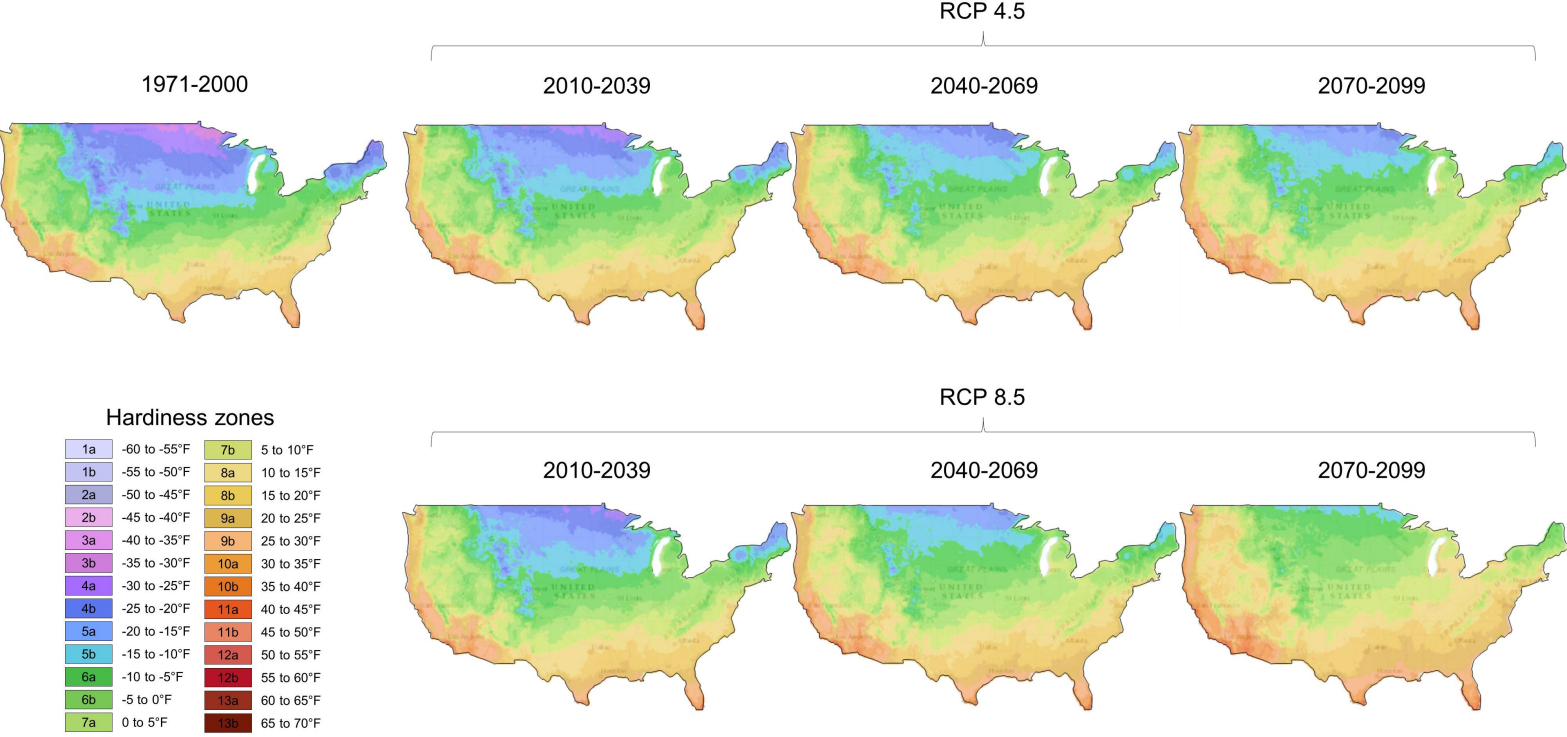
Mapping of future projections of plant hardiness zones in the US based on two different scenarios of greenhouse gas and aerosol emissions. Projecting future climate change involves assessing a number of different uncertainties. This figure shows plant hardiness changes in the US based on different greenhouse gas and aerosol emissions. Representative concentration pathways (RCPs) are possible future greenhouse gas and aerosol emissions scenarios. For example, RCP 4.5 represents a moderate emissions scenario in which emissions peak approximately in 2040 and then decline. RCP 8.5 represents a high emissions scenario called “business as usual”. Under RCP 4.5, temperatures are expected to rise between 1.1°C and 2.6°C (1.98°F and 4.68°F), while under RCP 8.5, global temperatures would rise between 2.6°C and 4.8°C (4.68°F and 8.64°F) by the end of the 21^st^ century. Note that the plant growing zones drastically change over the next several decades. Data were obtained from the Climate Toolbox (https://climatetoolbox.org/).

Rice, wheat, maize, and potato are the most widely consumed staple crops in the world, providing over 60% of the global food calories and playing a critical role in feeding the growing population. Given their importance for global food security, it is essential to understand how these crops will be impacted by climate change and to develop effective strategies for managing the associated risks. Singh et al. provided a comprehensive summary of the important wheat diseases in the US, covering their host range, symptoms, favorable conditions, disease management, and integrated disease management strategies, while considering the potential impacts of climate change in the coming decades. This information is critical for developing effective disease management practices that account for changing environmental conditions and ensure the sustainability of wheat production in the US and around the world.

High temperatures can exacerbate the effects of biotic stresses on plants. Recent studies have shown that cytosolic calcium signaling, including the calmodulin binding protein CBP60g, plays a critical role in ensuring the plant’s resilience to high temperatures (Kim et al., 2022) and in mediating the perception of both biotic and abiotic stress (Marcec et al., 2019; Xu et al., 2022). Carpentier et al. reviewed the current literature on the combined effects of biotic stress and temperature on calcium signaling. The authors highlighted several molecular components in calcium signaling that play an important role in plant responses against both biotic and abiotic stresses, concluding that calcium signaling is a critical component of the signaling networks that plants use to sense and respond to their environment. In addition to high temperature, Shen et al. tested the effect of elevated CO_2_ on rice plant yield (under future climate condition) and on the severity of one of the major threats, sheath blight disease. Interestingly, elevated CO_2_ levels could not compensate for the negative effect of elevated temperature on the yield. Adopting sound agronomic practices can mitigate disease risk and enhance crop yields under future climate change.

Nutrients play a crucial role in plant immunity by providing energy and building blocks for cells (Datnoff et al., 2007). Phosphorous is one of the three major nutrients that plants need for growth and reproduction. PHR1 is a crucial transcription factor that responds to phosphate starvation and facilitates metabolic reprogramming during phosphorous limitation, thereby connecting phosphate perception and signaling (Isidra-Arellano et al., 2021). In this issue, Wang et al. showed an additional role of PHR1 in Crowdipper, a perennial herb. The authors found that phosphate starvation increased alkaloid accumulation, which was dependent on PHR1. Their research provides valuable insights into the connection between secondary metabolism and nutrient supply.

Extracellular ATP is an important signal in plant growth, development, and stress responses (Tanaka et al., 2010; Tanaka et al., 2014). Matthus et al. provided an overview of extracellular ATP (eATP) signaling in phosphate deprivation. ATP is an important energy molecule that acts as a signaling molecule outside cells, especially when cells are damaged (Tanaka et al., 2014; Tanaka and Heil, 2021). Previously, the authors’ group discovered that eATP-mediated cytosolic calcium elevation was diminished under phosphate deprivation conditions (Matthus et al., 2019). In this issue, Matthus et al. further discussed a plausible mechanism and speculated that phosphate deprivation causes depletion of cytosolic ATP and eATP to compensate for phosphate nutrition, which may impair the activities of calcium channels/pumps and intracellular phosphorylation. This mechanism could apply to other signaling pathways for plant immune responses during Pi starvation.

Evolutionarily conserved genes are critical to plant physiology, especially in their response to stress. For example, mitogen-activated protein kinases (MPKs) are highly conserved across plants and crucially coordinates the plant stress response. Yu et al. performed a functional analysis of a moss gene under biotic and abiotic stresses using rice as a model system. BURP-like proteins originated from lower land plants and have diverged due to motif conversion. The authors showed that *PpBURP2* confers resistance to different abiotic stresses but also biotic stress of a bacterial disease. Notably, PpBURP2 was shown to directly regulate the MPK signaling pathway and plays an important role in tolerance to multiple abiotic and biotic stresses.

Although many research has largely focused on plant resistance genes (with coding RNAs), the role of noncoding RNAs (ncRNAs) in response to environmental stresses is becoming increasingly recognized. Li et al. presented recent updates on the regulatory roles of ncRNAs in tomato plants against a broad range of abiotic and biotic stresses. While substantial research has been done in Arabidopsis and legumes, the authors noted the importance of understanding the role and molecular mechanism of ncRNAs in response to stresses using tomato as a model plant, which will provide valuable insights for improving the adaptability of other vegetable crops against adverse stresses under future climate change.

The published studies in this issue cover important aspects of climate change challenges **–** abiotic stress and plant immunity. Climate change can directly impact the environmental factors in the plant disease triangle model and affect the host plant and pathogen through changes in environmental factors (**Figure 2**). For example, changes in temperature and precipitation can directly impact the life cycles and distribution of pathogens, which can in turn affect disease incidence and severity (IPPC Secretariat, 2021). Similarly, changes in the environment can directly affect the temperature and the availability of water, light, CO_2_, and nutrients in the soil, which can be abiotic stresses for plants and impact the growth and health of host plants as well as the host’s susceptibility to disease (Velásquez et al., 2018). More studies focusing on plant immunity under abiotic stresses are clearly needed to better understand how to increase plant resilience under climate change.

**Figure 2:**
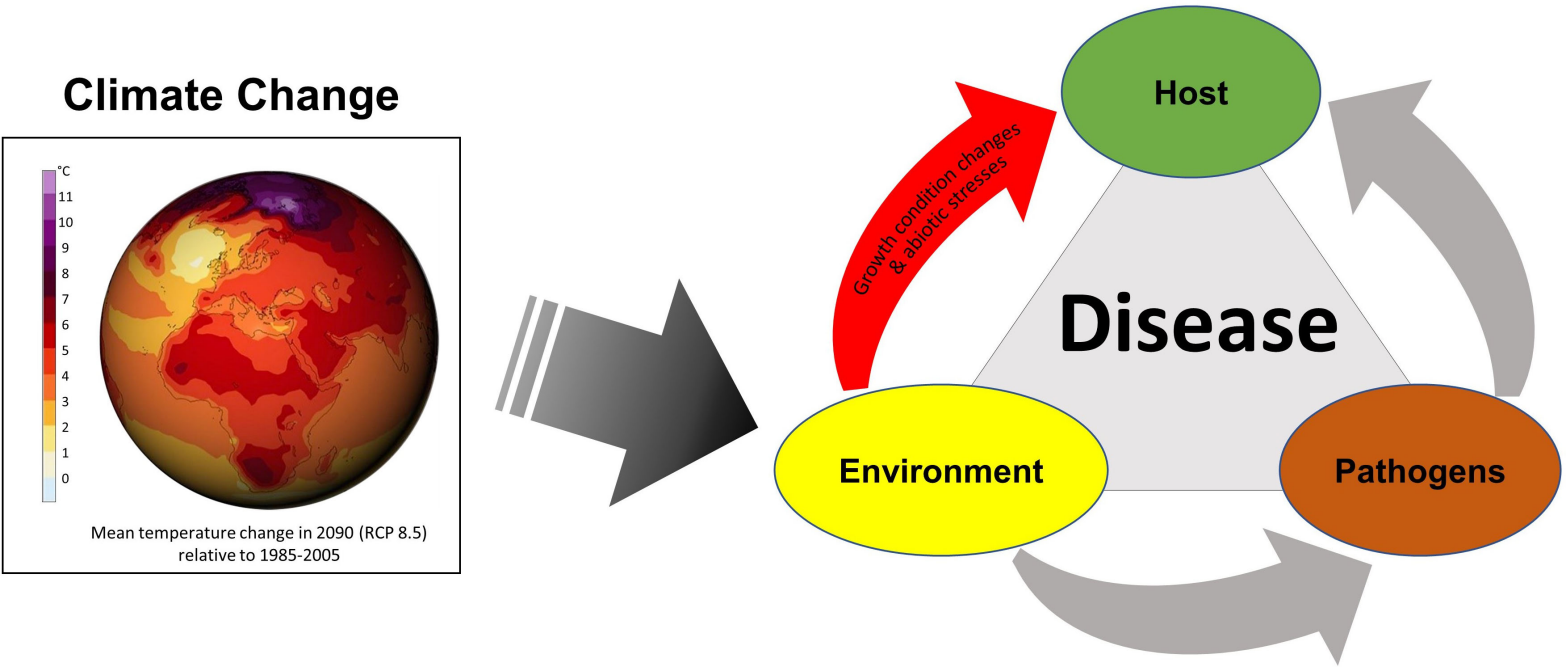
The plant disease triangle model with climate change. The disease triangle describes the interactions among the host plant, the pathogen, and the environment. These factors collectively contribute to the development and severity of plant diseases. Climate change can potentially cause significant impacts on each of these critical factors; notably, it can have a direct effect on the environment, including growth condition changes and abiotic stresses for plants, leading to modifications in the dynamics of plant diseases. It is important to consider the complex interactions between the three factors to gain a comprehensive understanding of how climate change might impact plant disease dynamics. Globe image source: DKRZ/MPI-M (https://www.dkrz.de/).

## Author contributions

All authors listed have made a substantial, direct, and intellectual contribution to the work and approved it for publication.

## References

[B1] Datnoff L. E. Elmer W. H. Huber D. M. (Eds.) (2007). Mineral nutrition and plant disease (St. Paul, MN: APS Press).

[B2] IPPC Secretariat (2021). Scientific review of the impact of climate change on plant pests – a global challenge to prevent and mitigate plant pest risks in agriculture, forestry and ecosystems (Rome, Italy: FAO on behalf of the IPPC Secretariat). doi: 10.4060/cb4769en

[B3] Isidra-Arellano M. C. Delaux P.-M. Valdés-López O. (2021). The phosphate starvation response system: its role in the regulation of plant–microbe interactions. Plant Cell Physiol. 62, 392–400. doi: 10.1093/pcp/pcab016 33515263

[B4] Kim J. H. Castroverde C. D. M. Huang S. Li C. Hilleary R. Seroka A.. (2022). Increasing the resilience of plant immunity to a warming climate. Nature 607, 339–344. doi: 10.1038/s41586-022-04902-y 35768511 PMC9279160

[B5] Mankin J. S. Seager R. Smerdon J. E. Cook B. I. Williams A. P. (2019). Mid-latitude freshwater availability reduced by projected vegetation responses to climate change. Nat. Geosci. 12, 983–988. doi: 10.1038/s41561-019-0480-x

[B6] Marcec M. J. Gilroy S. Poovaiah B. W. Tanaka K. (2019). Mutual interplay of Ca2+ and ROS signaling in plant immune response. Plant Sci 283, 343–354. doi: 10.1016/j.plantsci.2019.03.004 31128705

[B7] Matthus E. Wilkins K. A. Swarbreck S. M. Doddrell N. H. Doccula F. G. Costa A.. (2019). Phosphate starvation alters abiotic-Stress-Induced cytosolic free calcium increases in roots. Plant Physiol. 179, 1754–1767. doi: 10.1104/pp.18.01469 30696750 PMC6446763

[B8] Mora C. Caldwell I. R. Caldwell J. M. Fisher M. R. Genco B. M. Running S. W. (2015). Suitable days for plant growth disappear under projected climate change: potential human and biotic vulnerability. PloS Biol. 13, e1002167. doi: 10.1371/journal.pbio.1002167 26061091 PMC4465630

[B9] Tanaka K. Choi J. Cao Y. Stacey G. (2014). Extracellular ATP acts as a damage-associated molecular pattern (DAMP) signal in plants. Front. Plant Sci. 5. doi: 10.3389/fpls.2014.00446 PMC4153020 25232361

[B10] Tanaka K. Gilroy S. Jones A. M. Stacey G. (2010). Extracellular ATP signaling in plants. Trends Cell Biol. 20, 601–608. doi: 10.1016/j.tcb.2010.07.005 20817461 PMC4864069

[B11] Tanaka K. Heil M. (2021). Damage-associated molecular patterns (DAMPs) in plant innate immunity: applying the danger model and evolutionary perspectives. Annu. Rev. Phytopathol 59, 53–75. doi: 10.1146/annurev-phyto-082718-100146 33900789

[B12] Velásquez A. C. Castroverde C. D. M. He S. Y. (2018). Plant–pathogen warfare under changing climate conditions. Curr. Biol. 28, R619–R634. doi: 10.1016/j.cub.2018.03.054 29787730 PMC5967643

[B13] Xu T. Niu J. Jiang Z. (2022). Sensing mechanisms: calcium signaling mediated abiotic stress in plants. Front. Plant Sci. 13. doi: 10.3389/fpls.2022.925863 PMC9234572 35769297

